# Leveraging deep learning models to increase the representation of nomadic pastoralists in health campaigns and demographic surveillance

**DOI:** 10.1371/journal.pgph.0004018

**Published:** 2025-04-24

**Authors:** Benjamin Liu, Stace Maples, Jessie Kong, Francesco Fava, Nathaniel Jenson, Philemon Chelanga, Sergio Charles, James Hassell, Lance W. Robinson, Luke Glowacki, Michele Barry, Hannah B. Wild

**Affiliations:** 1 Department of Computer Science, Stanford University, Stanford, California, United States of America; 2 Department of Surgery, Stanford University, Stanford, California, United States of America; 3 Stanford Geospatial Center, Stanford University, Stanford, California United States of America; 4 Department of Environmental Science and Policy, University of Milan, Milan, Italy; 5 Global Academy of Agriculture and Food Systems, University of Edinburgh, Edinburgh, Scotland; 6 Agency for Inclusive Innovation Development, Nairobi, Kenya; 7 Global Health Program, Smithsonian’s National Zoo and Conservation Biology Institute, Washington, DC, United States of America; 8 Equitable Earth Initiative, Calgary, Canada; 9 Department of Anthropology, Boston University, Boston, Massachusetts, United States of America; 10 Center for Innovation in Global Health, Stanford University, Stanford, California, United States of America; 11 Department of Surgery, University of Washington, Seattle, Washington, United States of America; University of Cambridge, UNITED KINGDOM OF GREAT BRITAIN AND NORTHERN IRELAND

## Abstract

Nomadic pastoralists are systematically underrepresented in the planning of health services and frequently missed by health campaigns due to their mobility. Previous studies have developed novel geospatial methods to address these challenges but rely on manual techniques that are too time and resource-intensive to scale on a national or regional level. To address this gap, we developed a computer vision-based approach to automatically locate active nomadic pastoralist settlements from satellite imagery. We curated labeled datasets of satellite images capturing approximately 1,000 historically active settlements in the Omo Valley of Ethiopia and the Samburu County of Kenya to train and evaluate deep learning models, studying their robustness to low spatial resolutions and limits in labeled training data. Using a novel training strategy that leveraged public road and water infrastructure data, we closed performance gaps introduced by shortages in labeled settlement data. We deployed our best model on a region spanning 5,400 square kilometers in the Omo Valley of Ethiopia, resulting in the identification of historical settlements with a 270-fold reduction in manual review volume. Our work serves as a promising framework for automating the localization of nomadic pastoralist settlements at a national scale for health campaigns and demographic surveillance.

## 1. Introduction

Nomadic pastoralists migrate over large areas of remote terrain to support herds of livestock, which makes them susceptible to systematic underrepresentation in demographic surveys and census-reliant health campaigns [1]. Systematic underrepresentation of mobile populations can lead to biased national statistics and underfunding of pastoral regions. Furthermore, underrepresentation of sub-groups within pastoral populations, such as those that are most remote or mobile, can lead to imprecise and ineffective policy decisions by health officials working in these regions [2]. The implications of such bias are particularly significant considering the impact of climate change, food insecurity, conflict, and infectious diseases among nomadic pastoralist populations in numerous regions of Sub-Saharan Africa [3]. Previous studies have developed geospatial and remote sensing techniques to address these difficulties, including an approach capable of generating representative sampling strategies among nomadic populations using remote sensing data that was piloted and validated among the Nyangatom of Ethiopia’s South Omo Valley [2]. However, the pilot of this methodology relied on manual enumeration that limited scalability. In this study, we addressed these challenges by leveraging machine learning to automatically detect nomadic settlements from remotely sensed imagery.

In the last decade, the availability of remote sensing data and satellite imagery has increased substantially due to advancements in satellite missions and technology. The influx of this data has significantly expanded the scope and granularity of earth observation, providing unique opportunities to perform comprehensive mapping of local landmarks in a variety of spatial and temporal contexts [4]. Prominent satellite missions vary in their data collection capabilities along temporal, spatial, and spectral dimensions [5–7]. The costs of accessing data from these satellites are primarily determined by image resolution, sensor availability, and querying frequency, and all of these factors must be considerately weighed in designing humanitarian surveys [8].

In recent years, deep learning-based computer vision models have been applied widely to systematically address population-level issues by using remotely sensed imagery. Recent approaches have leveraged the wide breadth of temporal, spectral, and spatial data available to improve mapping efforts by making them increasingly scalable, robust, and accurate across diverse geographical settings [4]. For example, previous mapping studies have developed computer vision-based methods for successfully mapping informal settlements in Indonesia [9], wastewater treatment plants in Germany [8], and light-emitting infrastructure for socioeconomic studies [10]. To our knowledge, no previous studies have developed computer vision models for the mapping of nomadic pastoralist settlements nor studied the domain-specific challenges associated with applying such methods.

Here, we developed a novel computer vision model for the automatic localization of active nomadic pastoralist settlements from satellite imagery and evaluated the scalability of this method to a level of implementation that is compatible with national health campaigns. We showed that leveraging auxiliary settlement data such as roadway and waterway proximity can substantially improve model precision. Furthermore, we demonstrated that these strategies could augment performance in the face of limited training data, which is commonly observed in downstream model applications. To evaluate the robustness of our approach to regions with diverse geographical characteristics, we deployed our best model separately on pastoral regions in the South Omo Valley, Ethiopia and Samburu County, Kenya. This approach holds potential to improve inclusion of underrepresented nomadic pastoralist populations in health data, campaigns, and services. Moreover, it offers an accessible and generalizable framework for monitoring nomadic populations, creating potential opportunities to broaden integrated health efforts that facilitate disease surveillance, vaccination administration, and resource allocation in remote regions.

## 2. Methods

### 2.1. Dataset

**Fig 1:**
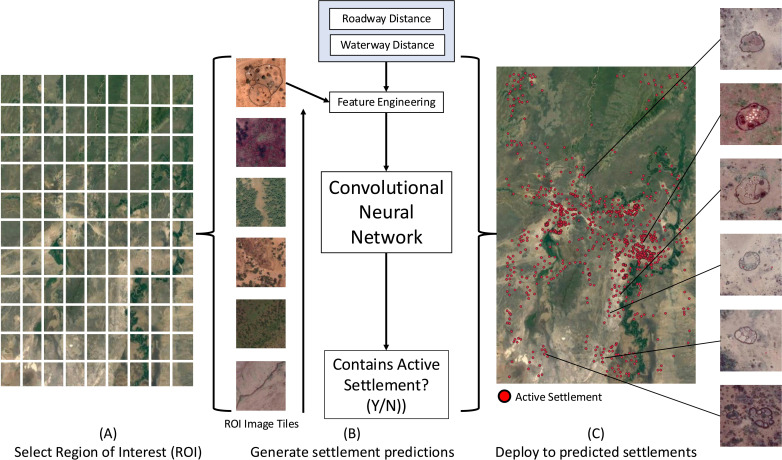
Gallery of images contained in our full dataset. Negative examples were sampled throughout continental Africa (M-T), near active settlements (U-X), and among inactive settlements (I-L). Active settlement examples occupied lands with diverse characteristics in both the Omo Valley of Ethiopia (A-D) and the Samburu County of Kenya (E-H). All displayed satellite images were sourced from the ESRI World Imagery basemap [11].

Two datasets of active settlements were separately derived from the Omo Valley of Ethiopia and the Samburu County of Kenya by using geographic bounds supplied by the ArcGIS hub and with feedback from experts at the International Livestock Research Institute (ILRI). Acknowledging that pastoralists practice mobility on a wide spectrum, we referred to “nomadic” to cover the full range of mobility. Although satellite images in the Omo Valley of Ethiopia and the Samburu County of Kenya differ in characteristics such as soil composition, terrain, occupied footprint, and settlement structure, common recognizable features can be analyzed to determine whether an image contains an active settlement. For example, active settlements are generally surrounded by darker circular enclosures, more distinctly defined in appearance, and feature the appearance of village huts and a lack of vegetation growth due to livestock grazing. In contrast, inactive settlements are characterized by burn marks and vegetation overgrowth (Fig 1).

To conduct an initial scan of high-density subareas for each dataset, we superimposed a Google Imagery satellite layer on top of existing geographical bounds and drew quadrilateral shapes to identify key settlement features such as village buildings and livestock enclosures. These features were then manually labeled as either active or inactive. We defined “high-density” based on the relative number of proximal pastoralist communities and settlements. Following this initial scan, areas within 1,000 meters from any given active settlement were queried and tiled into satellite images each covering 128m x 128m footprints. A similar procedure was completed for more distant areas to form a set of non-settlement image candidates, which we formally deemed as “negative examples.” Resulting satellite images entered a second round of manual labeling to ensure label correctness and account for temporal discrepancies between downloaded images and mosaiced images used in our initial scan. Images were obtained at various spatial resolutions as part of our investigation (Section 2.2.3). To increase the geographical diversity of our collection of negative examples, we randomly sampled geographical points from each of the 50 largest countries in Africa measured by land cover and additionally sampled random locations from distant areas of the country of interest. Publicly available shapefiles of roadway and waterway data in Ethiopia and Kenya were collected from the open-sourced Grid3 data repository [12] and augmented to our datasets in the form of infrastructure proximity-derived features for further experiments.

A detailed breakdown of both datasets used in our study can be seen in Table 1. The Samburu County dataset does not include geographical locations sampled outside of Kenya, as our model generalizability assessment is designed to replicate the constraints of local researchers, who are commonly restricted to training data in their region of interest. Since our study focuses heavily on locating active settlements from large regions of interest rather than determining whether a settlement is occupied, we excluded inactive settlements from our model experiments. However, as detailed in Section 3.4, we performed evaluations that included inactive settlements with our collection of negative examples to better understand the advantages of model pretraining.

**Table 1:** Composition of datasets used for model training and evaluation. “Random, Continental” refers to randomly sampled geographical locations across continental Africa, excluding the country of interest. “Random, National” refers to randomly sampled geographical locations in the country of interest. Satellite images containing active settlements were designated with “Positive” labels and all other images were designated with “Negative” labels. The training subset was used to learn model parameters, the validation subset was used to select model hyperparameters, and the test subset was used to perform a final evaluation. We used the Omo Valley dataset for all model experiments, while the Samburu County dataset was specifically used to assess model generalizability.

Dataset: Omo Valley, Ethiopia					
Data Type	Label	Train	Validation	Test	All Splits
Random, Continental	Negative	6906	1482	1469	9857
Random, National	Negative	1371	280	306	1957
Inactive Settlement^*^	Negative	1432	320	310	2062
Active Settlement	Positive	480	114	101	695
**Dataset**: Samburu County, Kenya					
Data Type	Label	Train	Validation	Test	Total
Random, National	Negative	1323	287	287	1807
Inactive Settlement^*^	Negative	324	69	70	463
Active Settlement	Positive	198	42	43	283

* Inactive settlements were solely used in our assessment of model generalizability (Section 3.4). The rationale for this choice is explained in Section 2.1.

### 2.2 Model Experiments

#### 2.2.1 Model Architecture and Training.

We trained deep convolutional neural networks (CNNs) to perform a binary classification task as a proxy for our overall mapping task. Specifically, we trained models with the objective of identifying whether a satellite image contains an active nomadic pastoralist settlement. Neural networks are computational models made of densely interconnected nodes designed to capture the complex non-linear relationships between inputs and outputs. CNNs are a special class of neural networks designed to process images by learning hierarchical features and spatial relationships using convolutional operations [13,14]. We tested prominent CNN and Transformer-based architectures including Vision Transformers [15], EfficientNet [16], DenseNet [17], ResNeXt [18], ResNet [14], and HRNet [19] with a focus on understanding the effect of macroscopic model architecture choices on performance (Table 2, S1 Text). In addition, we experimented with a wide array of training hyperparameters, including learning rate, optimizer choice, and regularization while keeping our architecture fixed. We observed that training an EfficientNet-B6 model with an Adam optimizer, batch size of 16, and a learning rate of 0.001 yielded an optimal performance within 40 epochs. Throughout our paper, we refer to this model trained under the given hyperparameters as our “best model.”

**Table 2:** Performances across prominent deep learning-based computer vision models on the settlement localization task using high-resolution satellite imagery. EfficientNet-B7 achieves the top performance across all metrics when trained under Adam optimization with a batch size of 16 and a learning rate of 0.001.

Architecture	F1	AUPRC	Precision at 95% Recall
HaloNet	0.910	0.958	0.727
ResNext-101	0.932	0.979	0.890
ResNet-200	0.916	0.968	0.848
HRNet	0.940	0.979	0.933
DenseNet-201	0.937	0.981	0.929
Vision Transformer	0.949	0.984	0.950
**EfficientNet-B7**	**0.957**	**0.984**	**0.968**

We encoded each image through our convolutional neural network to obtain dense feature maps, which are processed through a set of fully-connected multi perceptron (MLP) layers, yielding a probability score that an image contains an active settlement. In particular, let $\vec{x}\in {\mathbb{R}}^{H\times W\times C}$ represent an image with height H width W and number of channels C Likewise, let y denote its binary class label (i.e. whether the image contains an active settlement). Then for a given image $\vec{x}$, we model the probability that it belongs to an active settlement as


$$P\left(y=1\;|\;\vec{x};\theta \right)=h_{\theta }\left(\vec{x}\right)=f_{\phi }\left(E_{\psi }\left(\vec{x}\right)\right)$$

where $E_{\psi }:{\mathbb{R}}^{H\times W\times C}\to {\mathbb{R}}^{d}$ is a CNN encoder, $f_{\phi }:{\mathbb{R}}^{d}\to \left[0,1\right]$ is a fully-connected neural network that maps the d -dimensional latent representations to a probability, and $h_{\theta }:{\mathbb{R}}^{H\times W\times C}\to \left[0,1\right]$ denotes our full model as a composite function of $E_{\psi }$ and $f_{\phi }$. We leveraged the aforementioned hyperparameter settings in all experiments and trained our models under an unweighted binary cross entropy loss objective:


$$l\left(\theta \right)=\overset{n}{\underset{i=1}{\sum }}y^{\left(i\right)}\log\left(h_{\theta }\left({\vec{x}}^{\left(i\right)}\right)\right)+\left(1-y^{\left(i\right)}\right)\log\left(1-h_{\theta }\left({\vec{x}}^{\left(i\right)}\right)\right).$$

We then applied a threshold 0<α<1 among all probabilities to formally distinguish between images predicted to contain active settlements and those otherwise. The threshold used varies in the context of our specific evaluation. That is, we label an image as active if $P\left(y=1\text{|}\vec{x}\right)>\alpha$.

At the end of each epoch, we monitored our model’s area under the precision-recall curve (AUPRC) and precision at 0.95 recall on our validation set, using both metrics for model selection. We perform training and deployment using a single NVIDIA A4000 GPU. Each model completed training within two hours, and model deployment was performed in 24 hours.

#### 2.2.2 Evaluation metrics.

We used multiple metrics in the assessment of our model performance but focused on precision at high recall thresholds (i.e. 0.95) due to the prioritization of maximizing coverage of settlements over the sheer correctness of our predictions. We measured 1) the AUPRC to understand the progression of precision at various recall thresholds and 2) precision under high recall thresholds to assess the preciseness of our predictions while maximizing settlement coverage. We additionally recorded the top F1 score, which represents the harmonic mean between precision and recall in our evaluation.

#### 2.2.3 Spatial resolution ablation study.

To characterize the feasibility of training our models with different satellite imagery products, we analyzed the impact of spatial resolution on model performance by mirroring the resolution bounds of widely accessible satellite products. We initially labeled Google satellite images occupying a 128m x 128m footprint taken at a resolution of 0.5 m/pixel, as these parameters provided sufficient granularity to distinguish important settlement features. To evaluate different resolution settings, we started with 256 x 256 pixel images taken at a spatial resolution of 0.5 m/ pixel to maintain label consistency. We then scaled the images down such that the resulting dimensions rendered the images at resolutions of 1.0, 2.0, 3.0, 5.0, 8.0, and 10.0 m/ pixel. These specifications matched the resolution extents of prevalent satellite products including WorldView, GeoEye-1, QuickBird, Rapideye-5, Planet Scope, Sentinel-1, and Sentinel-2. Finally, we upsampled the modified images back to 256 x 256 pixels, thus creating a standalone change to spatial resolution while maintaining consistency in all other image parameters.

#### 2.2.4 Fusion of auxiliary data modeled with gaussian discriminant analysis.

We leveraged publicly available infrastructure data on roadways and waterways to develop a multi-modal learning approach to improve our model’s precision at high recall thresholds, particularly when faced with limits in labeled training data. We specifically leveraged the observation that active settlements tend to be located closer to roadways and waterways and furthermore, that the distributions of infrastructure proximities between active settlements and other points differ substantially. To capture these differences, we relied on a Gaussian discriminant analysis (GDA) procedure [20] to construct auxiliary features encoding information on distances to nearby infrastructure, such as roadways and waterways. The Maximum Likelihood Estimator (MLE) for GDA is asymptotically efficient [21], meaning it is well suited to handle limits in labeled training data, which we formally refer to as the low data regime. Specifically, we sampled subsets of our dataset separated by class to compute infrastructure proximity metrics and subsequently fitted multivariate Gaussian distributions to each class. We then sampled probabilities from these distributions to form our auxiliary features. We experimented with three different fusion strategies. Aux_(1)_ refers to adding auxiliary metric features to class logits $\hat{y}=f_{\phi }\left(E_{\psi }\left(\vec{x}\right)\right)$. Aux_(2)_ refers to appending auxiliary features to global-average-pooled, low-dimensional feature map embeddings $\vec{z}=E_{\psi }\left(\vec{x}\right)$. Aux_(1, 2)_ refers to simultaneously employing both strategies (S1 Fig).

More precisely, let D be a random variable representing the distance to a roadway. We take an image $\vec{x}$ and compute the distance between its geographical location and the nearest roadway. Then, we model the conditional, generative distribution of roadway distances D given the class label y as a univariate Gaussian, where *y* is modeled as a Bernoulli variable. In this case:


$$y∼Ber\left(\xi \right)$$

$$D|y=0∼N\left(\mu_{0},\sigma_{0}^{2}\right)$$

$$D|y=1∼N\left(\mu_{1},\sigma_{1}^{2}\right)$$

where $\mu_{0},\;\mu_{1}$ and $\sigma_{0}^{2},\;\sigma_{1}^{2}$ are the mean and variance parameters of the two generative distributions and *ξ* denotes the prevalence of active settlements in our dataset. We use maximum likelihood estimation to estimate the parameters $\hat{\mu_{0}}$, $\hat{\mu_{1}}$, $\hat{\sigma_{0}^{2}}$, $\hat{\sigma_{1}^{2}}$, and $\hat{\xi }$.

The objective is to take an arbitrary image $\vec{x}$, compute the distance $d^{\cdot }=d\left(\vec{x}\right)$ to the nearest roadway using its latitude and longitude coordinates, and determine the probability that the image belongs to a certain class. We use Bayes’ theorem to make this inference:


$$P\left(y=1\text{|}d^{\cdot }\right)=\frac{P\left(d^{\cdot }\text{|}y=1\right)P\left(y=1\right)}{P\left(d^{\cdot }\right)}$$

$$=\frac{P\left(d^{\cdot }\text{|}y=1\right)P\left(y=1\right)}{P\left(d^{\cdot }\text{|}y=0\right)P\left(y=0\right)+P\left(d^{\cdot }\text{|}y=1\right)P\left(y=1\right)}$$

whereby


$$P\left(d^{\cdot }\text{|}y=1\right)=\frac{1}{\sqrt{2\pi \hat{\sigma_{1}^{2}}}}\exp\left(-\frac{1}{2\hat{\sigma_{1}^{2}}}{\left(d^{\cdot }-\hat{\mu_{1}}\right)}^{2}\right),$$

$$P\left(d^{\cdot }\text{|}y=0\right)=\frac{1}{\sqrt{2\pi \hat{\sigma_{0}^{2}}}}\exp\left(-\frac{1}{2\hat{\sigma_{0}^{2}}}{\left(d^{\cdot }-\hat{\mu_{0}}\right)}^{2}\right),$$

$$P\left(y=1\right)=\hat{\xi },$$

$$P\left(y=0\right)=1-\hat{\xi }.$$

We leveraged these fitted probability density functions to compute probability features for each point in our full dataset, which excluded points used to fit our GDA model. An analogous procedure was performed to generate auxiliary features encoding waterway proximity. Computed probabilities, which were stratified by both label and infrastructure classes, were leveraged as individual features consistent with the Aux_(1)_, Aux_(2)_, and Aux_(1, 2)_ fusion strategies described above.

To assess the potential for this method to improve model robustness in low data regimes, we conducted ablation experiments where we trained models with datasets containing 25-100 labeled examples of active settlements. All experiments that we performed related to our GDA-based fusion approaches were performed with geographical locations exclusively sourced from Ethiopia to maintain consistency with available infrastructure data.

### 2.3 Model deployment

We deployed our best model on satellite images covering 5,400 square kilometers in the South Omo Valley region of Ethiopia at resolutions of 0.5 and 3.0 m/ pixel. Predicted probabilities were converted into binary predictions by using a threshold that obtained 0.95 recall of known locations. Positively predicted, adjacent images were merged by computing the centroid of their spatial union. Finally, a full manual review of predicted active settlements was performed to assess the overall precision of our model deployment. We supplemented this deployment exercise by fine-tuning and evaluating our best model on a second labeled dataset of active settlements in a defined region of the Samburu County of Kenya to assess the generalizability of our approach to settlement regions with differing characteristics.

## 3. Results

### 3.1 Spatial resolution ablation study

We observed that models trained with images at higher spatial resolutions outperformed those trained on images of lower resolutions (Table 3). Specifically, models trained at lower spatial resolution settings, including 5.0 m/pixel and 8.0 m/pixel achieved a precision at 95% recall of 0.734 and 0.627, respectively. Models trained with 3.0 m/pixel resolution imagery performed comparably to those of 0.5 m/pixel resolution imagery, displaying moderate drops in AUPRC and precision at 95% recall of approximately 0.01 and 0.13, respectively (Table 3).

**Table 3:** Model performances across different spatial resolution settings that capture the resolution bounds of prevalent satellite products.

Fixed Resolution	F1	AUPRC	Precision at 95% Recall
10.0 m/pixel	0.828	0.901	0.554
8.0 m/pixel	0.845	0.912	0.627
5.0 m/pixel	0.900	0.952	0.734
3.0 m/pixel	0.939	0.972	0.840
2.0 m/pixel	0.960	0.976	0.972
1.0 m/pixel	0.943	0.973	0.938
**0.5 m/pixel**	**0.957**	**0.984**	**0.968**

### 3.2 Fusion of auxiliary data modeled with gaussian discriminant analysis

We performed experiments to investigate the potential for publicly available auxiliary data to improve model precision at high recall thresholds. We observed that top-performing fusion approaches that separately leveraged waterway and roadway proximity data led to improvements in the precision at 95% recall by >0.13 and >0.12, respectively, relative to a non-fusion baseline. A GDA-based fusion approach that jointly incorporated waterway and roadway features outperformed our non-fusion baseline in precision at 95% recall by >0.03 (Table 4). In evaluating the direct contributions of GDA, we found that modeling distance features with our GDA approach led to gains in precision at 95% recall compared to fusing distance features directly (S1 Table).

**Table 4:** Model performance comparisons across GDA-based fusion approaches leveraging different classes of auxiliary data. Performances of the top model in each auxiliary data class measured by precision at 95% recall are bolded.

Auxiliary Data	Fusion Strategy	F1	AUPRC	Precision at 95% Recall
—	Baseline	0.925	0.964	0.774
Water	Aux_(1)_	0.921	0.968	0.799
	**Aux**_(2)_	**0.935**	**0.968**	**0.913**
	Aux_(1, 2)_	0.937	0.965	0.780
Road	**Aux**_(1)_	**0.932**	**0.964**	**0.900**
	Aux_(2)_	0.932	0.965	0.810
	Aux_(1, 2)_	0.931	0.967	0.881
Water + Road	**Aux**_(1)_	**0.926**	**0.960**	**0.809**
	Aux_(2)_	0.934	0.968	0.771
	Aux_(1, 2)_	0.938	0.966	0.751

### 3.3 Improving Model Robustness to Low Data Regimes

**Fig 2:**
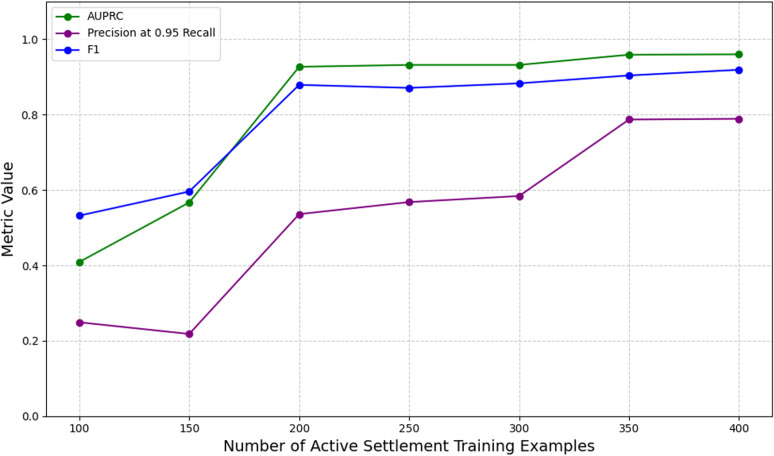
Graphical comparisons of model performance as a function of the numbers of active settlement examples provided during training. Model performance degrades substantially when fewer than 200 examples of active settlements are included in the training dataset.

**Fig 3:**
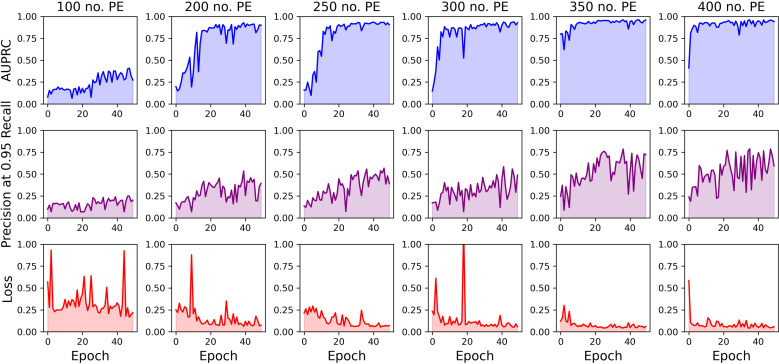
Graphical comparisons of model training progressions under exposure to different numbers of active settlement examples (no. PE) during training. Overall, training stability improves with higher numbers of active settlements in the training dataset.

We studied the performance of our models relative to the reduction of active settlement examples in our training dataset to understand and mitigate challenges associated with low data regimes. We initially observed that the performance of our baseline model with no auxiliary data supplements saw a substantial decrease across all metrics when the number of active settlement examples in the training dataset was lowered below a count of 200, with an AUPRC and precision at 95% recall of 0.567 and 0.218, respectively. Precision at 95% recall decreased notably when the number of active settlement examples in the training dataset was lowered below a count of 350 (Fig 2). These trends were upheld in observing the training progressions of our models under different data regimes. We observed that model training became increasingly unstable with a decrease in the number of active settlement examples included during training (Figs 2,3).

**Fig 4:**
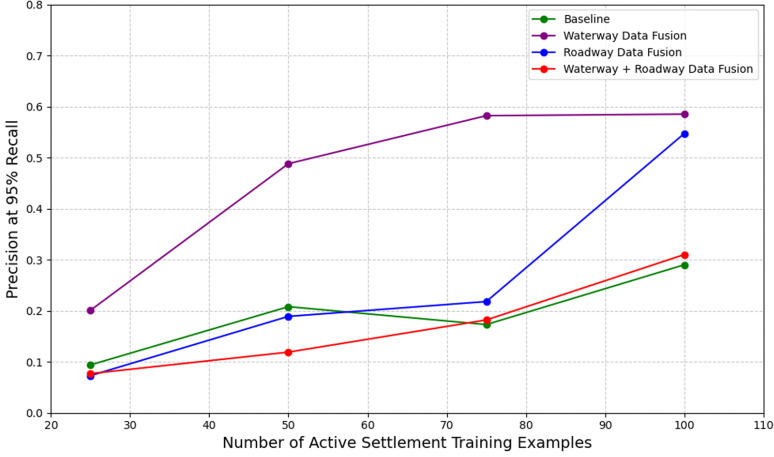
Graphical comparisons of model performance among top GDA-based fusion models in low data regimes relative to a non-fusion baseline model. Model performance in this graph is measured by precision at 95% recall. The GDA-based waterway fusion model outperformed its fusion counterpart models and its non-fusion baseline.

To address these challenges, we tested models that leveraged a GDA-based fusion approach with different classes of auxiliary data, including nearest waterway distance, nearest roadway distance, and a combination of both distance features. We observed that models trained with top-performing fusion approaches outperformed a baseline approach. Specifically, our top-performing waterway fusion model outperformed our baseline model across all low data regimes, with improvements of >0.10, >0.28, >0.40, and >0.29 in precision at 95% recall relative to training sets containing 25, 50, 75, and 100 active settlement examples, respectively. Our top-performing roadway fusion model outperformed our baseline model by >0.25 in precision at 95% recall when trained on 100 active settlement examples and performed comparably in all other settings. Similarly, the top-performing roadway-waterway fusion model performed comparably to our baseline model in all low data regimes (Fig 4).

### 3.4 Model deployment and generalizability

**Fig 5:**
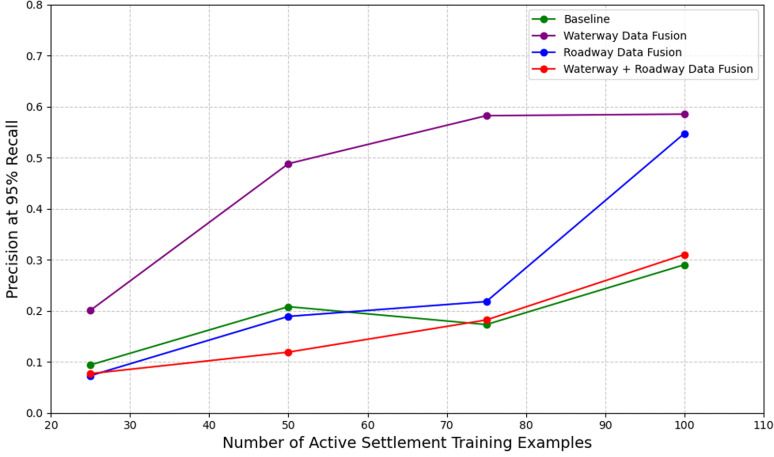
Maps of the Omo Valley deployment region in Ethiopia [ 11]. Active settlements (detections in red) tend to be located near roadways and waterways compared to a random sample of points in the area of interest (orange).

We deployed our best model on the full Omo Valley target region spanning 5,400 square kilometers at image resolutions of 0.5 m/pixel and 3.0 m/pixel (Fig 5). We subsequently performed a full manual review of active settlement predictions. Under image resolutions of 0.5 m/pixel and 3.0 m/pixel, our model achieved a precision at 95% recall of 0.71 and 0.61, respectively. Furthermore, we obtained a 270-fold search space reduction, reducing our number of active settlement candidates from 300,000 to ~1,100 for manual review.

To assess model generalizability, we finetuned and evaluated our best model using the dataset containing active settlements in the Samburu County of Kenya. Our baseline model trained solely on the Samburu County dataset achieved results comparable to our best model trained on the Omo Valley dataset (Table 5, Row 1). We observed that leveraging a model pretrained on the Omo Valley dataset improved precision at 95% recall by 0.03 compared to a randomly initialized baseline. When incorporating inactive settlements in our modeling task (Table 1), model pretraining results in an increase of 0.06 in precision at 95% recall, relative to a randomly initialized baseline (Table 5).

**Table 5:** Comparisons of model performance evaluated on our dataset from the Samburu Country of Kenya. Models pretrained on our Omo Valley settlement dataset outperformed randomly initialized models. This performance gap is widened when inactive settlements are incorporated as negative examples in the modeling task.

Inactive Settlements Included	Pretrained	F1	AUPRC	Precision at 95% Recall
No	No	0.96	0.98	0.95
No	Yes	0.96	0.98	0.98
Yes	No	0.88	0.92	0.74
Yes	Yes	0.96	0.87	0.80

## 4. Discussion

Our results demonstrate the potential for computer vision approaches to perform efficient and accurate localization of active nomadic pastoralist settlements from satellite imagery. Initial model experiments provided critical insights into the effect of model design choices and satellite image parameters on performance. We leveraged these insights to develop methods that use publicly available settlement auxiliary information in model training, achieving considerable performance improvements in low data regimes. We then deployed our best model over an extensive area of interest in the Omo Valley of Ethiopia, validating the scalability of our approach and its practical use in health and census campaigns. Collectively, our results demonstrate promising evidence of the potential for our methodology’s application in public health evaluations and health campaigns to increase inclusion and equity for underrepresented nomadic pastoralist populations.

Our experiments investigating the impact of spatial resolution on model training showed that overall, model performance gradually degrades at lower spatial resolution settings, which is consistent with previous studies [22]. Specifically, moderate drops in precision at 95% recall are observed at spatial resolution settings of 3.0 m/pixel and lower. Despite these moderate drops in performance, we observed that AUPRC is reasonably maintained above 0.9 over all spatial resolution settings and that even at the lowest tested spatial resolution setting, a precision at 95% recall above 0.5 is achieved. The results of these experiments provide important insight into the spatial resolutions of satellite images that are needed to achieve adequate levels of precision at high levels of recall on our task. Moreover, the nature of performance degradation over lower spatial resolutions provides valuable information about the level of spatial granularity that is needed to distinguish important settlement features and hence, make accurate predictions. Public health researchers can leverage this information to make informed decisions on the spatial scope of their studies, adjusting for available resources and personnel.

Our experiments leveraging publicly available infrastructure proximity data reveal that auxiliary information about active settlements can be successfully leveraged to improve model performance, boosting precision at 95% recall by as much as >0.13. The observation that improvements are observed after leveraging waterway and roadway proximity data, separately, indicate that the observed effect is potentially generalizable to other types of auxiliary information for which the behavior of associated distributions is substantially different among classes of interest. There was no clear dominance of a single fusion strategy in our experiments, as some fusion strategies achieved higher performances relative to our model baseline under different classes of auxiliary data. This suggests that the effectiveness of a given fusion strategy may depend on the nature of auxiliary feature differences captured in the class-stratified distributions, although additional studies must be performed to study these trends. In further ablation experiments, we observed that notable performance improvements offered by GDA-based fusion approaches were dampened at spatial resolutions that were lower than 5.0 m/ pixel. Future work should investigate this observation and provide insight into whether these approaches are dependent on sufficiently granular depictions of important image features. The effectiveness of our GDA-based fusion approaches may offer important insights for other machine learning mapping studies that seek to leverage differences in associated auxiliary information to improve the precision of their vision-based predictions.

In follow-up experiments where we tested the ability for GDA-based fusion approaches to alleviate challenges posed by low data regimes, we observed that augmenting our baseline model with a waterway distance-based fusion approach led to substantial improvements to precision at 95% recall over all low data settings. These observations offer important insights for the development of computer vision models for active settlement localization, as it is conventionally difficult to obtain active settlement labels for model development. Common reasons for this trend include the low density of nomadic pastoralist settlements, sparse nature of settlement distributions, and limitations on personnel, compute power, and image acquisition. Furthermore, the success of these approaches in low data regimes suggests that strong priors on image classes can be effectively leveraged to close performance gaps presented by data limitations. This observation may be useful to adjacent machine learning mapping studies with constraints in accessible data. Future work should incorporate the domain expertise of public health officials to investigate other auxiliary priors that can be leveraged and quantify the generalizability limitations of models trained in these conditions.

The successful deployment of our models over a 5,400 square-kilometer region in Omo Valley, Ethiopia demonstrated the real-world effectiveness of our approach in performing a timely, automatic mapping of active settlements. Gaps in model performance that were observed between model development and model deployment are comparable to differences observed in similar machine learning mapping studies for energy infrastructure [23] and can possibly be attributed to the geographical diversity of the Omo Valley region and corresponding differences in active settlement appearances that were not captured in our training dataset. Modest differences in performance between models trained on images obtained at 1.0 and 3.0 m/ pixel resolution suggest that large-scale deployments can be successfully carried out with satellite images obtained at lower spatial resolutions of 3.0 m/ pixel. This finding has important implications for public health campaigns that are considering the integration of these methods, since it expands the number of available satellite products for consideration by alleviating pressure on image acquisition costs. Overall, we found that deploying our model resulted in a substantial 270-fold reduction of the search space for active settlements, reducing our number of active settlement candidates from 300,000 to ~1,100 for manual review. This search space reduction gives credence to the expanded flexibility and feasibility that our approach offers for analyzing large areas of interest that would be impractical to analyze manually. Crucially, our approach addresses scalability concerns noted in several related studies [24–26] and with further development, shows promise in being implemented at a level consistent with national health campaigns. Supplementary ablation experiments that were conducted on data derived from the Samburu County of Kenya offer strong evidence that our approach can be applied to regions with diverse geographical characteristics, and performance improvements attained from pretraining suggest that our models are learning generalizable features that encode important, shared characteristics of active settlements. The latter was further supported by test-time saliency maps, which commonly highlighted universal settlement features, such as fencing, kraals, and huts (S2 Fig).

Our study has several important implications for existing global public health efforts focused on nomadic pastoralists and other mobile populations, particularly in remote regions. By addressing key bottlenecks in demographic surveys and census methodologies that have previously limited representative sampling of this population, our approach provides a more systematic and scalable way to capture data on mobile groups including nomadic pastoralists. Traditional survey techniques often rely heavily on random sampling techniques, which are inherently limited in coverage, or on manual enumeration methods that are both time and resource-intensive [26–28]. Additionally, existing strategies require expert knowledge to be effectively deployed, further restricting their application in remote and under-resourced settings [2,29]. By leveraging deep learning for settlement identification, our method may significantly alleviate these constraints. This would not only enable demographic surveys to reach previously inaccessible locations at scale but also allow for more frequent and timely data collection. We emphasize that such approaches should be used in a context-appropriate manner and implemented in partnership with local collaborators to ensure sensitivity to local dynamics, particularly in conflict-affected settings.

Due to the cross-cutting nature of this methodological challenge across diverse global public health studies, our approach could support both research and service delivery among nomadic pastoralists across several key domains. In the context of climate change, our method could streamline existing efforts to assess how pastoralists are being affected by shifting environmental conditions and adapting to these challenges [30]. Similarly, given the ability to conduct full censuses of settlement locations at scale, our methodology holds potential to aid in evaluating food security dynamics, which frequently intersect both with climate stressors and conflict dynamics [31–33]. From a One Health perspective, this framework could augment the study of infectious disease transmission within and between pastoralist communities, particularly in relation to zoonotic and enteric parasitic diseases, which remain a significant yet understudied risk factor [3]. This methodology also holds potential for the design of public health campaigns as well as strategies to assess and the uptake of critical health services, ensuring that vaccinations, maternal health interventions, and disease surveillance programs more effectively reach mobile populations [34–36]. Collectively, these improvements could lead to better-informed health policy decisions for nomadic pastoralists and support integrated health frameworks such as One Health [37,38].

In future work, integration of our approach with community engagement and participatory mapping efforts to provide primers on pastoralist mobility patterns should be explored. Since the early 1990s, diverse methods for participatory mapping have become commonplace in development practice [39], specifically for pastoralist communities [40–42]. Participatory mapping has the potential to offer more detailed information on pastoralist mobility patterns compared to analysis of remote sensing data alone. For example, it could assist in locating individuals, such as hired herders and family members, who are migrating with herds away from household locations. Combining our approach with participatory mapping methods and traditional ecological knowledge will require caution and keen awareness of local power dynamics, however. For example, in surveying herding destinations that the state or other powerful actors disapprove of, it will be important to carefully manage sensitive information that may lead to conflict [40].

There were several limitations to this study. First, active settlement labels were designated based on inspecting high-resolution satellite imagery. Although physical aerial markers of settlement activity exist, we were not able to obtain visual evidence on the ground to validate our judgements. In future work, we aim to collaborate with field experts and local mapping authorities to obtain ground truth and rectify real-world gaps in our labeling criteria. Second, while our model substantially reduces the volume of active settlement locations that must be screened, it still necessitates a manual review of location candidates. This requirement may pose a limitation to public health efforts due to a lack of resources and personnel. Third, due to the relatively small spatial footprints of pastoralist settlements, we relied on high-resolution satellite imagery in our study, which is expensive and inaccessible on a global scale. Although we demonstrated that our approach can be feasibly applied with satellite images at spatial resolutions as low as 10.0 m/pixel, we hope to perform further studies to quantify model performance constraints at more coarse resolution settings, such as those offered by Landsat-9 at 30 m/pixel [43] and Sentinel-2 at 20 m/pixel [44].

## 5. Conclusion

In this study, we developed a computer vision-based approach for the localization of active nomadic pastoralist settlements from satellite imagery. We highlighted key considerations that are important to the integration and development of these models in health campaigns and demographic surveillance. Specifically, by performing a comprehensive evaluation of key model properties, including architectures, spatial resolutions, and robustness, we found that an EfficientNet-B6 model could perform optimally at spatial resolutions of 3.0 m/ pixel with as few as 200 active settlements in training. Moreover, we developed a novel approach for settlement localization by leveraging public infrastructure data and showed that modeling these features with Gaussian Discriminant Analysis (GDA) could substantially augment performance in low data regimes, which is uniquely advantageous for global health applications. We then demonstrated successful large-scale deployments of these models in diverse geographical areas of interest in Ethiopia and Kenya. Overall, our approach provides a strong framework for the integration of computational remote sensing in large-scale, demographic health campaigns. The generalizability of our method provides new opportunities to scale the study of nomadic pastoralists across several public health domains, such as climate change, food insecurity, and disease transmission, thereby strengthening integrated health frameworks for mobile populations.

## Supporting information

S1 Text Description of model architecture and training. (DOCX)

S1 Table Comparisons of model performance across different fusion approaches, ablating the use of GDA to process auxiliary distance features. “None” indicates the use of normalized distances without GDA-based preprocessing. Performances of the top model in each auxiliary data class measured by precision at 95% recall are bolded.
(XLSX)

S1 Fig Visual summary of GDA-based fusion model architectures. Auxiliary distance features in our study were defined either as the distance to the nearest waterway or roadway. All displayed satellite images were sourced from the ESRI World Imagery basemap [11].
(TIFF)

S2 Fig Gallery of active settlement predictions and saliency map counterparts from our test set. Across active settlements in Ethiopia (A-C) and Kenya (D-F), saliency maps focused heavily on universal settlement features such as fencing boundaries, livestock enclosures, and village huts. All displayed satellite images were sourced from the ESRI World Imagery basemap [11]. (TIFF)
