# Correction: Leveraging deep learning models to increase the representation of nomadic pastoralists in health campaigns and demographic surveillance

**DOI:** 10.1371/journal.pgph.0005757

**Published:** 2025-12-31

**Authors:** Benjamin Liu, Stace Maples, Jessie Kong, Francesco Fava, Nathaniel Jensen, Philemon Chelanga, Sergio Charles, James Hassell, Lance W. Robinson, Luke Glowacki, Michele Barry, Hannah B. Wild

The fifth author’s name is spelled incorrectly. The correct name is: Nathaniel Jensen. The correct citation is: Liu B, Maples S, Kong J, Fava F, Jensen N, Chelanga P, et al. (2025) Leveraging deep learning models to increase the representation of nomadic pastoralists in health campaigns and demographic surveillance. PLOS Glob Public Health 5(4): e0004018. https://doi.org/10.1371/journal.pgph.0004018
