# Supplementary material for: Leveraging deep learning models to increase the representation of nomadic pastoralists in health campaigns and demographic surveillance
Source: PLOS Glob Public Health. 2025 Apr 24;5(4):e0004018. doi: 10.1371/journal.pgph.0004018 (PMC12021161; doi:10.1371/journal.pgph.0004018)
Supplement: S1 Text — (DOCX) [file pgph.0004018.s001.docx]

S1 Text: Model Architecture and Training

We tested prominent CNN and Transformer-based architectures. ResNet introduced residual connections to stabilize training in deep neural networks and served as the foundation for deep learning-based computer vision models [1]. ResNeXt built on these designs with the invention of cardinality, allowing for parallel learning paths that augmented model capacity [2]. DenseNet similarly introduced the notion of dense connectivity within deep networks and thus encouraged parameter efficiency and improved gradient flows with more compact model representations [3]. EfficientNet achieved a breakthrough in efficiency by scaling dimensions proportionally, which made it an ideal candidate under strict computational requirements [4]. HRNet, with its multi-resolution approach, excels in preserving spatial details and can be useful in fine-grained tasks such as image segmentation or classification tasks that involve subtle distinctions [5]. Finally, Vision Transformers leverage self-attention to capture long-range dependencies, making them ideal for recognizing complex patterns, albeit under extensive computational requirements [6]. Each architecture offers distinct advantages, which we must weigh considerately with the nature of our task and availability of resources in expected application settings.

References

1. He K, Zhang X, Ren S, Sun J. Deep Residual Learning for Image Recognition [Internet]. arXiv; 2015 [cited 2023 May 1]. Available from: http://arxiv.org/abs/1512.03385

2. Xie S, Girshick R, Dollár P, Tu Z, He K. Aggregated residual transformations for deep neural networks. In: Proceedings of the IEEE conference on computer vision and pattern recognition [Internet]. 2017 [cited 2024 Jul 28]. p. 1492–500. Available from: http://openaccess.thecvf.com/content_cvpr_2017/html/Xie_Aggregated_Residual_Transformations_CVPR_2017_paper.html

3. Huang G, Liu Z, Van Der Maaten L, Weinberger KQ. Densely Connected Convolutional Networks. In: 2017 IEEE Conference on Computer Vision and Pattern Recognition (CVPR) [Internet]. Honolulu, HI: IEEE; 2017 [cited 2023 May 1]. p. 2261–9. Available from: https://ieeexplore.ieee.org/document/8099726/

4. Tan M, Le Q. EfficientNet: Rethinking Model Scaling for Convolutional Neural Networks. In: Proceedings of the 36th International Conference on Machine Learning [Internet]. PMLR; 2019 [cited 2023 May 1]. p. 6105–14. Available from: https://proceedings.mlr.press/v97/tan19a.html

5. Wang J, Sun K, Cheng T, Jiang B, Deng C, Zhao Y, Liu D, Mu Y, Tan M, Wang X. Deep high-resolution representation learning for visual recognition. IEEE transactions on pattern analysis and machine intelligence. 2020;43(10):3349–64.

6. Khan S, Naseer M, Hayat M, Zamir SW, Khan FS, Shah M. Transformers in Vision: A Survey. ACM Comput Surv. 2022 Jan 31;54(10s):1–41.
