# Supplementary figures and images for: Leveraging deep learning models to increase the representation of nomadic pastoralists in health campaigns and demographic surveillance
Source: PLOS Glob Public Health. 2025 Apr 24;5(4):e0004018. doi: 10.1371/journal.pgph.0004018 (PMC12021161; doi:10.1371/journal.pgph.0004018)

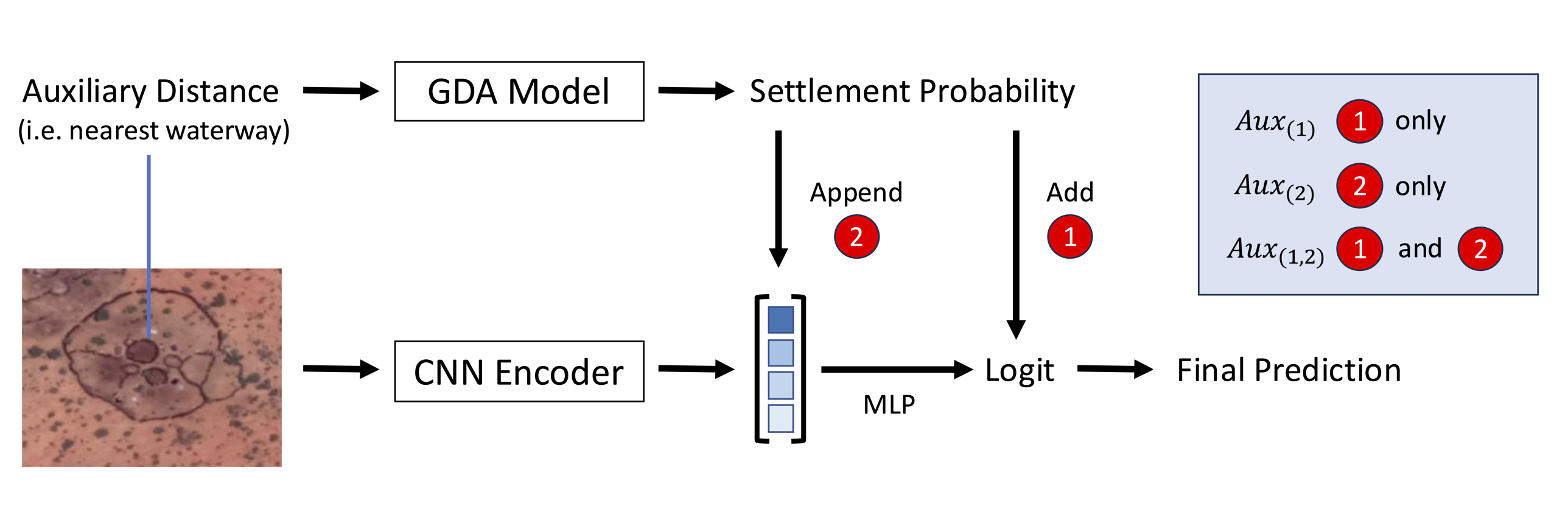

Supplement: S1 Fig — Auxiliary distance features in our study were defined either as the distance to the nearest waterway or roadway. All displayed satellite images were sourced from the ESRI World Imagery basemap [11]. (TIFF) [file pgph.0004018.s003.tiff]
